# Relapse prevention for addictive behaviors

**DOI:** 10.1186/1747-597X-6-17

**Published:** 2011-07-19

**Authors:** Christian S Hendershot, Katie Witkiewitz, William H George, G Alan Marlatt

**Affiliations:** 1 Centre for Addiction and Mental Health, 33 Russell St., Toronto, ON, M5S 2S1, Canada; 2 Department of Psychiatry, University of Toronto, 250 College St., Toronto, ON M5T 1R8, Canada; 3 Department of Psychology, Washington State University, 14204 NE Salmon Creek Ave, Vancouver, WA, 98686, USA; 4 Department of Psychology, University of Washington, Box 351525, Seattle, WA 98195, USA

**Keywords:** Alcohol, cognitive-behavioral skills training, continuing care, drug use, psychosocial intervention, substance use treatment

## Abstract

The Relapse Prevention (RP) model has been a mainstay of addictions theory and treatment since its introduction three decades ago. This paper provides an overview and update of RP for addictive behaviors with a focus on developments over the last decade (2000-2010). Major treatment outcome studies and meta-analyses are summarized, as are selected empirical findings relevant to the tenets of the RP model. Notable advances in RP in the last decade include the introduction of a reformulated cognitive-behavioral model of relapse, the application of advanced statistical methods to model relapse in large randomized trials, and the development of mindfulness-based relapse prevention. We also review the emergent literature on genetic correlates of relapse following pharmacological and behavioral treatments. The continued influence of RP is evidenced by its integration in most cognitive-behavioral substance use interventions. However, the tendency to subsume RP within other treatment modalities has posed a barrier to systematic evaluation of the RP model. Overall, RP remains an influential cognitive-behavioral framework that can inform both theoretical and clinical approaches to understanding and facilitating behavior change.

## Introduction

Relapse poses a fundamental barrier to the treatment of addictive behaviors by representing the modal outcome of behavior change efforts [1-3]. For instance, twelve-month relapse rates following alcohol or tobacco cessation attempts generally range from 80-95% [1,4] and evidence suggests comparable relapse trajectories across various classes of substance use [1,5,6]. Preventing relapse or minimizing its extent is therefore a prerequisite for any attempt to facilitate successful, long-term changes in addictive behaviors.

Relapse prevention (RP) is a tertiary intervention strategy for reducing the likelihood and severity of relapse following the cessation or reduction of problematic behaviors. Three decades since its introduction [7], the RP model remains an influential cognitive-behavioral approach in the treatment and study of addictions. The aim of this paper is to provide readers with an update on empirical and applied developments related to RP, with a primary focus on events spanning the last decade (2000-2010). We begin with a concise overview of the historical and theoretical foundations of the RP model and a brief summary of clinical intervention strategies. Next, we review the major theoretical, methodological and applied developments related to RP in the last decade. Specific emphasis is placed on the reformulated cognitive-behavioral model of relapse [8] as a basis for hypothesizing and studying dynamic aspects of the relapse process. In reviewing empirical findings we focus on major treatment outcome studies, meta-analyses, and selected results that coincide with underlying tenets of the RP model. We conclude by noting critiques of the RP model and summarizing current and future directions in studying and preventing relapse.

This paper extends recent reviews of the RP literature [1,8-10] in several ways. Most notably, we provide a recent update of the RP literature by focusing primarily on studies conducted within the last decade. We also provide updated reviews of research areas that have seen notable growth in the last few years; in particular, the application of advanced statistical modeling techniques to large treatment outcome datasets and the development of mindfulness-based relapse prevention. Additionally, we review the nascent but rapidly growing literature on genetic predictors of relapse following substance use interventions. In focusing exclusively on addictive behaviors (for which the RP model was initially conceived) we forego a discussion of RP as it relates to various other behavioral domains (e.g., sexual offending, depression, diet and exercise) and refer readers to other sources for updates on the growing range of RP applications [8,11].

### Definitions of relapse and relapse prevention

The terms "relapse" and "relapse prevention" have seen evolving definitions, complicating efforts to review and evaluate the relevant literature. Definitions of relapse are varied, ranging from a dichotomous treatment outcome to an ongoing, transitional process [8,12,13]. Overall, a large volume of research has yielded no consensus operational definition of the term [14,15]. For present purposes we define *relapse *as a setback that occurs during the behavior change process, such that progress toward the initiation or maintenance of a behavior change goal (e.g., abstinence from drug use) is interrupted by a reversion to the target behavior. We also take the perspective that relapse is best conceptualized as a dynamic, ongoing process rather than a discrete or terminal event (e.g., [1,8,10]).

Definitions of RP have also evolved considerably, due largely to the increasingly broad adoption of RP approaches in various treatment contexts. Though the phrase "relapse prevention" was initially coined to denote a specific clinical intervention program [7,16], RP strategies are now integral to most psychosocial treatments for substance use [17], including many of the most widely disseminated interventions (e.g., [18-20]). The National Registry of Evidence-based Programs and Practices, maintained by the U.S. Substance Abuse and Mental Health Services Administration (SAMHSA), includes listings for numerous empirically supported interventions with "relapse prevention" as a descriptor or primary treatment objective (http://www.nrepp.samhsa.gov). Thus, RP has in many ways evolved into an umbrella term encompassing most skills-based treatments that emphasize cognitive-behavioral skills building and coping responses. While attesting to the broad influence of the RP model, the diffuse application of RP approaches also tends to complicate efforts to define RP-based treatments and evaluate their overall efficacy (e.g., [21]). In the present review we emphasize Marlatt's RP model [7,16] and its more recent iteration [8] when discussing the theoretical basis of RP. By necessity, our literature review also includes studies that do not explicitly espouse the RP model, but that are relevant nonetheless to its predictions.

### Marlatt's relapse prevention model: Historical foundations and overview

The RP model developed by Marlatt [7,16] provides both a conceptual framework for understanding relapse and a set of treatment strategies designed to limit relapse likelihood and severity. Because detailed accounts of the model's historical background and theoretical underpinnings have been published elsewhere (e.g., [16,22,23]), we limit the current discussion to a concise review of the model's history, core concepts and clinical applications.

Based on the cognitive-behavioral model of relapse, RP was initially conceived as an outgrowth and augmentation of traditional behavioral approaches to studying and treating addictions. The evolution of cognitive-behavioral theories of substance use brought notable changes in the conceptualization of relapse, many of which departed from traditional (e.g., disease-based) models of addiction. For instance, whereas traditional models often attribute relapse to endogenous factors like cravings or withdrawal--construed as symptoms of an underlying disease state--cognitive-behavioral theories emphasize contextual factors (e.g., environmental stimuli and cognitive processes) as proximal relapse antecedents. Cognitive-behavioral theories also diverged from disease models in rejecting the notion of relapse as a dichotomous outcome. Rather than being viewed as a state or endpoint signaling treatment failure, relapse is considered a fluctuating process that begins prior to and extends beyond the return to the target behavior [8,24]. From this standpoint, an initial return to the target behavior after a period of volitional abstinence (a *lapse*) is seen not as a dead end, but as a fork in the road. While a lapse might prompt a full-blown relapse, another possible outcome is that the problem behavior is corrected and the desired behavior re-instantiated--an event referred to as *prolapse*. A critical implication is that rather than signaling a failure in the behavior change process, lapses can be considered temporary setbacks that present opportunities for new learning to occur. In viewing relapse as a common (albeit undesirable) event, emphasizing contextual antecedents over internal causes, and distinguishing relapse from treatment failure, the RP model introduced a comprehensive, flexible and optimistic alternative to traditional approaches.

**Figure 1:**
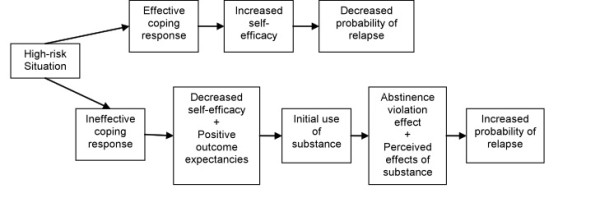
Original cognitive-behavioral model of relapse (Marlatt & Gordon, 1985)

Marlatt's original RP model is depicted in Figure 1. A basic assumption is that relapse events are immediately preceded by a *high-risk situation*, broadly defined as any context that confers vulnerability for engaging in the target behavior. Examples of high-risk contexts include emotional or cognitive states (e.g., negative affect, diminished self-efficacy), environmental contingencies (e.g., conditioned drug cues), or physiological states (e.g., acute withdrawal). Although some high-risk situations appear nearly universal across addictive behaviors (e.g., negative affect; [25]), high-risk situations are likely to vary across behaviors, across individuals, and within the same individual over time [10]. Whether a high-risk situation culminates in a lapse depends largely on the individual's capacity to enact an effective *coping response*--defined as any cognitive or behavioral compensatory strategy that reduces the likelihood of lapsing.

Central to the RP model is the role of cognitive factors in determining relapse liability. For example, successful navigation of high-risk situations may increase self-efficacy (one's perceived capacity to cope with an impending situation or task; [26]), in turn decreasing relapse probability. Conversely, a return to the target behavior can undermine self-efficacy, increasing the risk of future lapses. Outcome expectancies (anticipated effects of substance use; [27]) also figure prominently in the RP model. Additionally, attitudes or beliefs about the causes and meaning of a lapse may influence whether a full relapse ensues. Viewing a lapse as a personal failure may lead to feelings of guilt and abandonment of the behavior change goal [24]. This reaction, termed the Abstinence Violation Effect (AVE; [16]), is considered more likely when one holds a dichotomous view of relapse and/or neglects to consider situational explanations for lapsing. In sum, the RP framework emphasizes high-risk contexts, coping responses, self-efficacy, affect, expectancies and the AVE as primary relapse antecedents.

Implicit in the RP approach is that the initiation and maintenance of behavior change represent separate processes governed by unique contingencies [12,28]. Thus, specific cognitive and behavioral strategies are often necessary to maintain initial treatment gains and minimize relapse likelihood following initial behavior change. RP strategies fall into two broad categories: specific intervention techniques, often designed to help the patient anticipate and cope with high-risk situations, and global self-control approaches, intended to reduce relapse risk by promoting positive lifestyle change. An essential starting point in treatment is a thorough assessment of the client's substance use patterns, high-risk situations and coping skills. Other important assessment targets include the client's self-efficacy, outcome expectancies, readiness to change, and concomitant factors that could complicate treatment (e.g., comorbid disorders, neuropsychological deficits). Using high-risk situations as a starting point, the clinician works backward to identify immediate precipitants and distal lifestyle factors related to relapse, and forward to evaluate coping responses [16,24]. Ideally, this approach helps clients to recognize high-risk situations as discriminative stimuli signaling relapse risk, as well as to identify cognitive and behavioral strategies to obviate these situations or minimize their impact. Examples of specific intervention strategies include enhancing self-efficacy (e.g., by setting achievable behavioral goals) and eliminating myths and placebo effects (e.g., by challenging misperceptions about the effects of substance use).

The client's appraisal of lapses also serves as a pivotal intervention point in that these reactions can determine whether a lapse escalates or desists. Establishing lapse management plans can aid the client in self-correcting soon after a slip, and cognitive restructuring can help clients to re-frame the meaning of the event and minimize the AVE [24]. A final emphasis in the RP approach is the global intervention of lifestyle balancing, designed to target more pervasive factors that can function as relapse antecedents. For example, clients can be encouraged to increase their engagement in rewarding or stress-reducing activities into their daily routine. Success in these areas may enhance self-efficacy, in turn reducing relapse risk. Overall, the RP model is characterized by a highly ideographic treatment approach, a contrast to the "one size fits all" approach typical of certain traditional treatments. Moreover, an emphasis on post-treatment maintenance renders RP a useful adjunct to various treatment modalities (e.g., cognitive-behavioral, twelve step programs, pharmacotherapy), irrespective of the strategies used to enact initial behavior change.

## Developments in Relapse Prevention: 2000-2010

The last decade has seen numerous developments in the RP literature, including the publication of *Relapse Prevention, Second Edition *[29] and its companion text, *Assessment of Addictive Behaviors, Second Edition *[30]. The following sections provide an overview of major theoretical, empirical and applied advances related to RP over the last decade.

### The reformulated cognitive-behavioral model of relapse

**Figure 2:**
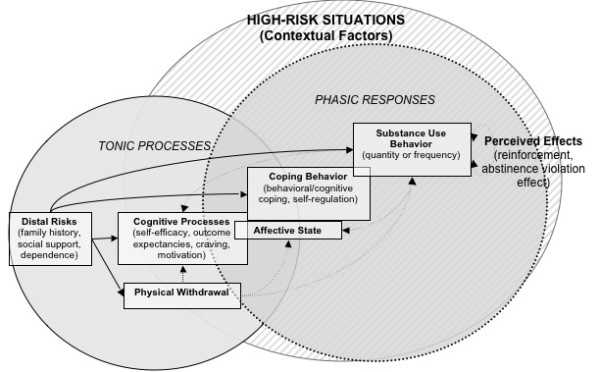
Revised cognitive-behavioral model of relapse (Witkiewitz & Marlatt, 2004)

Efforts to develop, test and refine theoretical models are critical to enhancing the understanding and prevention of relapse [1,2,14]. A major development in this respect was the reformulation of Marlatt's cognitive-behavioral relapse model to place greater emphasis on dynamic relapse processes [8]. Whereas most theories presume linear relationships among constructs, the reformulated model (Figure 2) views relapse as a complex, nonlinear process in which various factors act jointly and interactively to affect relapse timing and severity. Similar to the original RP model, the dynamic model centers on the high-risk situation. Against this backdrop, both tonic (stable) and phasic (transient) influences interact to determine relapse likelihood. Tonic processes include *distal risks--*stable background factors that determine an individual's "set point" or initial threshold for relapse [8,31]. Personality, genetic or familial risk factors, drug sensitivity/metabolism and physical withdrawal profiles are examples of distal variables that could influence relapse liability a priori. Tonic processes also include cognitive factors that show relative stability over time, such as drug-related outcome expectancies, global self-efficacy, and personal beliefs about abstinence or relapse. Whereas tonic processes may dictate initial susceptibility to relapse, its occurrence is determined largely by *phasic responses*--proximal or transient factors that serve to actuate (or prevent) a lapse. Phasic responses include cognitive and affective processes that can fluctuate across time and contexts--such as urges/cravings, mood, or transient changes in outcome expectancies, self-efficacy, or motivation. Additionally, momentary coping responses can serve as phasic events that may determine whether a high-risk situation culminates in a lapse. Substance use and its immediate consequences (e.g., impaired decision-making, the AVE) are additional phasic processes that are set into motion once a lapse occurs. Thus, whereas tonic processes can determine *who *is vulnerable for relapse, phasic processes determine *when *relapse occurs [8,31].

A key feature of the dynamic model is its emphasis on the complex interplay between tonic and phasic processes. As indicated in Figure 2, distal risks may influence relapse either directly or indirectly (via phasic processes). The model also predicts feedback loops among hypothesized constructs. For instance, the return to substance use can have reciprocal effects on the same cognitive or affective factors (motivation, mood, self-efficacy) that contributed to the lapse. Lapses may also evoke physiological (e.g., alleviation of withdrawal) and/or cognitive (e.g., the AVE) responses that in turn determine whether use escalates or desists. The dynamic model further emphasizes the importance of nonlinear relationships and timing/sequencing of events. For instance, in a high-risk context, a slight and momentary drop in self-efficacy could have a disproportionate impact on other relapse antecedents (negative affect, expectancies) [8]. Furthermore, the strength of proximal influences on relapse may vary based on distal risk factors, with these relationships becoming increasingly nonlinear as distal risk increases [31]. For example, one could imagine a situation whereby a client who is relatively committed to abstinence from alcohol encounters a neighbor who invites the client into his home for a drink. Feeling somewhat uncomfortable with the offer the client might experience a slight decrease in self-efficacy, which cascades into positive outcome expectancies about the potential effects of having a drink as well as feelings of shame or guilt about saying no to his neighbor's offer. Importantly, this client might not have ever considered such an invitation as a high-risk situation, yet various contextual factors may interact to predict a lapse.

The dynamic model of relapse assumes that relapse can take the form of sudden and unexpected returns to the target behavior. This concurs not only with clinical observations, but also with contemporary learning models stipulating that recently modified behavior is inherently unstable and easily swayed by context [32]. While maintaining its footing in cognitive-behavioral theory, the revised model also draws from nonlinear dynamical systems theory (NDST) and catastrophe theory, both approaches for understanding the operation of complex systems [10,33]. Detailed discussions of relapse in relation to NDST and catastrophe theory are available elsewhere [10,31,34].

### Empirical findings relevant to the RP model

The empirical literature on relapse in addictions has grown substantially over the past decade. Because the volume and scope of this work precludes an exhaustive review, the following section summarizes a select body of findings reflective of the literature and relevant to RP theory. The studies reviewed focus primarily on alcohol and tobacco cessation, however, it should be noted that RP principles have been applied to an increasing range of addictive behaviors [10,11].

## Systematic reviews and large-scale treatment outcome studies

The first comprehensive review of RP treatment outcome studies was Carroll's [35] descriptive account of 24 interventions focusing on substance use. This review found consistent support for the superiority of RP over no treatment, inconsistent support for its superiority over discussion control conditions, and consistent support that RP was equally efficacious to other active treatments. Carroll concluded that RP, though not consistently superior to other active treatments, showed particular promise in three areas: reducing relapse severity, enhancing durability of treatment gains, and matching treatment strategies to client characteristics. RP also showed delayed emergence effects in some studies, suggesting that it may outperform other treatments during the maintenance stage of behavior change [35].

Subsequently, a meta-analysis evaluated 26 RP treatment outcome studies totaling 9,504 participants [36]. The authors examined two primary outcomes (substance use and psychosocial functioning) and several treatment moderators. Effect sizes indicated that RP was generally successful in reducing substance use (*r *= .14) and improving psychosocial functioning (*r *= .48), consistent with its purpose as both a specific and global intervention approach. Moderation analyses suggested that RP was consistently efficacious across treatment modalities (individual vs. group) and settings (inpatient vs. outpatient). RP was most effective for reducing alcohol and polysubstance use and less effective for tobacco and cocaine use--a contrast to Carroll's [35] finding of comparable efficacy across drug classes. In addition, RP was more effective when delivered in conjunction with pharmacotherapy, when compared to wait-list (vs. active) comparison conditions, and when outcomes were assessed soon after treatment. Though some findings were considered tentative due to sample sizes, the authors concluded that RP was broadly efficacious [36].

McCrady [37] conducted a comprehensive review of 62 alcohol treatment outcome studies comprising 13 psychosocial approaches. Two approaches--RP and brief intervention--qualified as empirically validated treatments based on established criteria. Interestingly, Miller and Wilbourne's [21] review of clinical trials, which evaluated the efficacy of 46 different alcohol treatments, ranked "relapse prevention" as 35^th ^out of 46 treatments based on methodological quality and treatment effect sizes. However, many of the treatments ranked in the top 10 (including brief interventions, social skills training, community reinforcement, behavior contracting, behavioral marital therapy, and self-monitoring) incorporate RP components. These two reviews highlighted the increasing difficulty of classifying interventions as specifically constituting RP, given that many treatments for substance use disorders (e.g., cognitive behavioral treatment (CBT)) are based on the cognitive behavioral model of relapse developed for RP [16]. One of the key distinctions between CBT and RP in the field is that the term "CBT" is more often used to describe stand-alone primary treatments that are based on the cognitive-behavioral model, whereas RP is more often used to describe aftercare treatment. Given that CBT is often used as a stand-alone treatment it may include additional components that are not always provided in RP. For example, the CBT intervention developed in Project MATCH [18] (described below) equated to RP with respect to the core sessions, but it also included elective sessions that are not typically a focus in RP (e.g., job-seeking skills, family involvement).

An increasing number of large-scale trials have allowed for statistically powerful evaluations of psychosocial interventions for alcohol use. Project MATCH [18] evaluated the efficacy of three interventions--Motivational Enhancement Therapy (MET), Twelve-Step Facilitation (TSF), and Cognitive Behavioral Therapy (CBT)--for treating alcohol dependence. The CBT intervention was a skills-based treatment containing elements of RP. Spanning nine data collection sites and following over 1700 participants for up to three years, Project MATCH was the largest psychotherapy trial conducted to that point. Multiple matching hypotheses were proposed in evaluating differential treatment efficacy as a function of theoretically relevant client attributes. Primary analyses supported only one of sixteen matching hypotheses: outpatients lower in psychiatric severity fared better in TSF than in CBT during the year following treatment [18]. Although primary analyses provided relatively little support for tailoring alcohol treatments based on specific client attributes, matching effects have been identified in subsequent analyses (described in more detail later).

Since 2005 an ongoing Cochrane review has evaluated RP for smoking cessation [38,39]. As of 2009, meta-analyses had found no support for the efficacy of skills-based RP approaches in preventing relapse to smoking [38]. However, a recent re-analysis of these trials yielded different results [40]. The re-analysis stratified behavioral interventions based on specific intervention content while also imposing stricter analytic criteria regarding the length of follow-up assessments. In these analyses, CBT/RP-based self-help interventions showed a significant overall effect in increasing long-term abstinence (pooled OR: 1.52, 95% CI: 1.15 - 2.01, based on 3 studies) and group counseling showed significant short-term efficacy (pooled OR: 2.55, 95% CI: 1.58 - 4.11, based on 2 studies). There was limited evidence for the efficacy of other specific behavioral treatments, although there was general support for the efficacy of pharmacological treatments [40].

Recently, Magill and Ray [41] conducted a meta-analysis of 53 controlled trials of CBT for substance use disorders. As noted by the authors, the CBT studies evaluated in their review were based primarily on the RP model [29]. Overall, the results were consistent with the review conducted by Irvin and colleagues, in that the authors concluded that 58% of individuals who received CBT had better outcomes than those in comparison conditions. In contrast with the findings of Irvin and colleagues [36], Magill and Ray [41] found that CBT was most effective for individuals with marijuana use disorders.

## Recent findings in support of RP model components

The following section reviews selected empirical findings that support or coincide with tenets of the RP model. Sections are organized in accordance with major model constructs. Because the scope of this literature precludes an exhaustive review, we highlight select findings that are relevant to the main tenets of the RP model, in particular those that coincide with predictions of the reformulated model of relapse.

### Self-efficacy

Self-efficacy (SE), the perceived ability to enact a given behavior in a specified context [26], is a principal determinant of health behavior according to social-cognitive theories. In fact, some theories view SE as the final common pathway to relapse [42]. Although SE is proposed as a fluctuating and dynamic construct [26], most studies rely on static measures of SE, preventing evaluation of within-person changes over time or contexts [43]. Shiffman, Gwaltney and colleagues have used ecological momentary assessment (EMA; [44]) to examine temporal variations in SE in relation to smoking relapse. Findings from these studies suggested that participants' SE was lower on the day before a lapse, and that lower SE in the days following a lapse in turn predicted progression to relapse [43,45]. One study [46] reported increases in daily SE during abstinent intervals, perhaps indicating mounting confidence as treatment goals were maintained [45].

In the first study to examine relapse in relation to phasic changes in SE [46], researchers reported results that appear consistent with the dynamic model of relapse. During a smoking cessation attempt, participants reported on SE, negative affect and urges at random intervals. Findings indicated nonlinear relationships between SE and urges, such that momentary SE decreased linearly as urges increased but dropped abruptly as urges peaked. Moreover, this finding appeared attributable to individual differences in baseline (tonic) levels of SE. When urge and negative affect were low, individuals with low, intermediate or high baseline SE were similar in their momentary SE ratings. However, these groups' momentary ratings diverged significantly at high levels of urges and negative affect, such that those with low baseline SE had large drops in momentary SE in the face of increasingly challenging situations. These findings support that higher distal risk can result in bifurcations (divergent patterns) of behavior as the level of proximal risk factors increase, consistent with predictions from nonlinear dynamic systems theory [31].

A recent meta-analysis evaluated the association of SE with smoking relapse [47]. The review included 54 studies that assessed prospective associations of SE and smoking during a quit attempt. A major finding concerned differential effect sizes based on the timing of SE assessments: the negative association of SE with likelihood of future smoking represented a small effect (*d *= -.21) when SE was assessed prior to the quit attempt, but a medium effect (*d *= -.47) when SE was assessed after the quit day. The authors concluded that, given the centrality of SE to most cognitive-behavioral models of relapse, the association of SE with cessation was weaker than would be expected (i.e., SE accounted for roughly 2% of the variance in treatment outcome following initial abstinence). The findings also suggested that SE should ideally be measured after the cessation attempt, and that controlling for concurrent smoking is critical when examining SE in relation to prospective relapse [47]. Finally, in analyses from a cross-national study of the natural history of smoking cessation, researchers examined self-efficacy in relation to relapse rates across an extended time period [48,49]. Results indicated that self-efficacy increased with cumulative abstinence and correlated negatively with urges, consistent with RP theory. Also, higher self-efficacy consistently predicted lower relapse rates across time and partly mediated the association of perceived benefits of smoking with relapse events [48,49].

### Outcome expectancies

Outcome expectancies (anticipated outcomes of a given behavior or situation) are central to the RP model and have been studied extensively in the domain of alcohol use [27]. In theory, expectancies are shaped by various tonic risk factors (e.g., environment, culture, personality, genetics) and mediate these antecedent influences on drinking [27]. Research supports that expectancies could partly mediate influences such as personality factors [50], genetic variations [51,52], and negative affect [53] on drinking. Outcome expectancies could also be involved as a phasic response to situational factors. In the first study to examine how daily fluctuations in expectancies predict relapse [45], researchers assessed positive outcome expectancies for smoking (POEs) among participants during a tobacco cessation attempt. Lower POEs on the quit day were associated with greater abstinence likelihood, and POEs decreased in the days following the quit day. Lapses were associated with higher POEs on the preceding day, and in the days following a lapse those who avoided a full relapse showed decreases in POEs whereas those who relapsed did not [45]. These results suggest that outcome expectancies might play a role in predicting relapse as both a tonic and phasic risk factor.

Expectancy research has recently started examining the influences of implicit cognitive processes, generally defined as those operating automatically or outside conscious awareness [54,55]. Recent reviews provide a convincing rationale for the putative role of implicit processes in addictive behaviors and relapse [54,56,57]. Implicit measures of alcohol-related cognitions can discriminate among light and heavy drinkers [58] and predict drinking above and beyond explicit measures [59]. One study found that smokers' attentional bias to tobacco cues predicted early lapses during a quit attempt, but this relationship was not evident among people receiving nicotine replacement therapy, who showed reduced attention to cues [60].

Initial evidence suggests that implicit measures of expectancies are correlated with relapse outcomes, as demonstrated in one study of heroin users [61]. In another recent study, researchers trained participants in attentional bias modification (ABM) during inpatient treatment for alcohol dependence and measured relapse over the course of three months post-treatment [62]. Relative to a control condition, ABM resulted in significantly improved ability to disengage from alcohol-related stimuli during attentional bias tasks. While incidence of relapse did not differ between groups, the ABM group showed a significantly longer time to first heavy drinking day compared to the control group. Additionally, the intervention had no effect on subjective measures of craving, suggesting the possibility that intervention effects may have been specific to implicit cognitive processes [62]. Overall, research on implicit cognitions stands to enhance understanding of dynamic relapse processes and could ultimately aid in predicting lapses during high-risk situations.

### Withdrawal

Withdrawal tendencies can develop early in the course of addiction [25] and symptom profiles can vary based on stable intra-individual factors [63], suggesting the involvement of tonic processes. Despite serving as a chief diagnostic criterion, withdrawal often does not predict relapse, perhaps partly explaining its de-emphasis in contemporary motivational models of addiction [64]. However, recent studies show that withdrawal profiles are complex, multi-faceted and idiosyncratic, and that in the context of fine-grained analyses withdrawal indeed can predict relapse [64,65]. Such findings have contributed to renewed interest in negative reinforcement models of drug use [63].

Although withdrawal is usually viewed as a physiological process, recent theory emphasizes the importance of behavioral withdrawal processes [66]. Whereas physiological withdrawal symptoms tend to abate in the days or weeks following drug cessation, the unavailability of a conditioned *behavioral *coping response (e.g., the ritual of drug administration) may leave the former user ill-equipped to cope with ongoing stressors, thus exacerbating and/or prolonging symptoms [66]. Current theory and research indicate that physiological components of drug withdrawal may be motivationally inert, with the core motivational constituent of withdrawal being negative affect [25,66]. Thus, examining withdrawal in relation to relapse may only prove useful to the extent that negative affect is assessed adequately [64].

### Negative affect

A large literature attests to the role of negative affect (NA) in the etiology and maintenance of addictive behaviors. NA is consistently cited as a relapse trigger in retrospective reports (e.g., [67,68]), although participants might sometimes misattribute lapses to negative mood states[15]. In one study, individuals who were unable to sustain a smoking cessation attempt for more than 24 hours (compared to those with a sustained quit attempt) reported greater depressive symptoms and NA in response to stress and displayed less perseverance during experimental stress inductions [69]. Supporting the dynamic influence of NA on relapse, Shiffman and Waters [70] found that smoking lapses were not associated with NA in the preceding days, but were associated with rising NA in the hours leading up to a lapse. Evidence further suggests that negative affect can promote positive outcome expectancies [53] or undermine situational self-efficacy [71], outcomes which could in turn promote a lapse. Moreover, Baker and colleagues propose that high levels of negative affect can interfere with controlled cognitive processes, such that adaptive coping and decision-making may be undermined as negative affect peaks [25]. Witkiewitz and Villarroel [72] found that drinking rates following treatment were significantly associated with current and prior changes in negative affect and changes in negative affect were significantly associated with current and prior changes in drinking state (effect size range = 0.13 (small) to 0.33 (medium)). Overall, the results showed that individuals who reported higher negative affect or increased negative affect over time had the highest probability of heavy and frequent drinking following treatment, and had a near-zero probability of transitioning to moderate drinking. Heavier and more frequent alcohol use predicted a greater probability of high negative affect and increased negative affect over time.

Knowledge about the role of NA in drinking behavior has benefited from daily process studies in which participants provide regular reports of mood and drinking. Such studies have shown that both positive and negative moods show close temporal links to alcohol use [73]. One study [74] found evidence suggesting a feedback cycle of mood and drinking whereby elevated daily levels of NA predicted alcohol use, which in turn predicted spikes in NA. These findings were moderated by gender, social context, and time of week. Other studies have similarly found that relationships between daily events and/or mood and drinking can vary based on intraindividual or situational factors [73], suggesting dynamic interplay between these influences.

### Self-control and coping responses

Strengthening coping skills is a goal of virtually all cognitive-behavioral interventions for substance use [75]. Several studies have used EMA to examine coping responses in real time. One study [76] found that momentary coping differentiated smoking lapses from temptations, such that coping responses were reported in 91% of successful resists vs. 24% of lapses. Shiffman and colleagues [68] found that restorative coping following a smoking lapse decreased the likelihood of a second lapse the same day. Exactly how coping responses reduce the likelihood of lapsing remains unclear. One study found that momentary coping reduced urges among smokers, suggesting a possible mechanism [76]. Some studies find that the number of coping responses is more predictive of lapses than the specific type of coping used [76,77]. However, despite findings that coping can prevent lapses there is scant evidence to show that skills-based interventions in fact lead to improved coping [75].

Some researchers propose that the self-control required to maintain behavior change strains motivational resources, and that this "fatigue" can undermine subsequent self-control efforts [78]. Consistent with this idea, EMA studies have shown that social drinkers report greater alcohol consumption and violations of self-imposed drinking limits on days when self-control demands are high [79]. Limit violations were predictive of responses consistent with the AVE the following day, and greater distress about violations in turn predicted greater drinking [80]. Findings also suggested that these relationships varied based on individual differences, suggesting the interplay of static and dynamic factors in AVE responses. Evidence further suggests that practicing routine acts of self-control can reduce short-term incidence of relapse. For instance, Muraven [81] conducted a study in which participants were randomly assigned to practice small acts self-control acts on a daily basis for two weeks prior to a smoking cessation attempt. Compared to a control group, those who practiced self-control showed significantly longer time until relapse in the following month.

## Emerging topics in relapse and relapse prevention

### Using nonlinear methods to model relapse

A key contribution of the reformulated relapse model is to highlight the need for non-traditional assessment and analytic approaches to better understand relapse. Most studies of relapse rely on statistical methods that assume continuous linear relationships, but these methods may be inadequate for studying a behavior characterized by discontinuity and abrupt changes [33]. Consistent with the tenets of the reformulated RP model, several studies suggest advantages of nonlinear statistical approaches for studying relapse.

In one study, researchers used catastrophe models to examine proximal and distal predictors of post-treatment drinking among individuals with alcohol use disorders [31]. Catastrophe models accounted for more than double the amount of variance in drinking than that predicted by linear models. Similar results have been found using the much larger Project MATCH dataset [33]. Two additional recent analyses of the MATCH dataset showed that nonlinear approaches can detect processes that may go unobserved in the context of linear models. Witkiewitz and colleagues [34,82] used catastrophe modeling and latent growth mixture modeling to re-assess two of the matching hypotheses that were not supported in the original study--that individuals low in baseline self-efficacy would respond more favorably to cognitive-behavioral therapy (CBT) than motivational enhancement therapy (MET) and that individuals low in baseline motivation would respond more favorably to MET than CBT [18]. In the first study [34], catastrophe models provided the best fit to the data, and latent growth analyses confirmed the predicted interaction: frequent drinkers with low initial self-efficacy had better outcomes in CBT than in MET, while those high in self-efficacy fared better in MET. Similarly, a second study [82] found that individuals in the outpatient arm of Project MATCH with low motivation to change at baseline who were assigned to MET had better outcomes than those assigned to CBT. The authors also found a treatment by gender by alcohol dependence severity interaction in support of the matching hypothesis, whereby females with low baseline motivation and males with lower levels of alcohol dependence and low baseline motivation who received MET as an aftercare treatment had better outcomes than those who were assigned to receive CBT as an aftercare treatment.

### Genetic influences on treatment response and relapse

The last decade has seen a marked increase in the number of human molecular genetic studies in medical and behavioral research, due largely to rapid technological advances in genotyping platforms, decreasing cost of molecular analyses, and the advent of genome-wide association studies (GWAS). Not surprisingly, molecular genetic approaches have increasingly been incorporated in treatment outcome studies, allowing novel opportunities to study biological influences on relapse. Given the rapid growth in this area, we allocate a portion of this review to discussing initial evidence for genetic associations with relapse. Specifically, we focus on recent, representative findings from studies evaluating candidate single nucleotide polymorphisms (SNPs) as moderators of response to substance use interventions. It is important to note that these studies were not designed to evaluate specific components of the RP model, nor do these studies explicitly espouse the RP model. Also, many studies have focused solely on pharmacological interventions, and are therefore not directly related to the RP model. However, we review these findings in order to illustrate the scope of initial efforts to include genetic predictors in treatment studies that examine relapse as a clinical outcome. These findings may be informative for researchers who wish to incorporate genetic variables in future studies of relapse and relapse prevention.

Broadly speaking, there are at least three primary contexts in which genetic variation could influence liability for relapse during or following treatment. First, in the context of pharmacotherapy interventions, relevant genetic variations can impact drug pharmacokinetics or pharmacodynamics, thereby moderating treatment response (pharmacogenetics). Second, the likelihood of abstinence following a behavioral or pharmacological intervention can be moderated by genetic influences on metabolic processes, receptor activity/expression, and/or incentive value specific to the addictive substance in question. For instance, SNPs with functional implications for relevant neurotransmitter or metabolic pathways can influence the reward value of marijuana (e.g., *FAAH; CNR1*); nicotine (e.g., *CYP2A6*, *CHRNB2*, *CHRNA4*); and alcohol (*ALDH2, ADH1B*), while others show potential for influencing the incentive value of multiple drugs (e.g., *ANKK1*; *DRD4*; *OPRM1*). Third, variants implicated in broad traits relevant for addictive behaviors--for instance, executive cognitive functioning (e.g., *COMT*) or externalizing traits (e.g., *GABRA2*, *DRD4*)--could influence relapse proneness via general neurobehavioral mechanisms, irrespective of drug class or treatment modality. As summarized below, preliminary empirical support exists for each of these possibilities.

Genetic influences on relapse have been studied most extensively in the context of pharmacogenetics, with the bulk of studies focusing on nicotine dependence (for recent reviews see [83,84]). Several candidate polymorphisms have been examined in response to smoking cessation treatments, especially nicotine replacement therapy (NRT) and bupropion [84]. The catechol-O-methyltransferase (COMT) Val158Met polymorphism, established as predicting variability in prefrontal dopamine levels, has been evaluated in relation to smoking cessation in several studies. Independent trials of NRT have found cessation rates to differ based on *COMT *genotype [85-87]. A polymorphism in the nicotinic acetylcholine ß2 receptor gene (*CHRNB2*) has been associated with length of abstinence and withdrawal symptoms during bupropion treatment [88] and with relapse rates and ability to quit on the target day during NRT [89]. One bupropion trial found that *DRD2 *variations predicted withdrawal symptoms, medication response and time to relapse [90]. In a study of the mu-opioid receptor (*OPRM1*) Asn40/Asp40 variant during NRT, those with the Asp40 variant had higher rates of abstinence and reduced negative affect compared to Asn40 individuals [91]. Additionally, post-hoc analyses indicated that Asp40 carriers were more likely to regain abstinence following a lapse, suggesting a possible role of the genotype in predicting prolapse.

The most promising pharmacogenetic evidence in alcohol interventions concerns the *OPRM1 *A118G polymorphism as a moderator of clinical response to naltrexone (NTX). An initial retrospective analysis of NTX trials found that *OPRM1 *influenced treatment response, such that individuals with the Asp40 variant (G allele) receiving NTX had a longer time until the first heavy drinking day and were half as likely to relapse compared to those homozygous for the Asn40 variant (A allele) [92]. This finding was later extended in the COMBINE study, such that G carriers showed a greater proportion of days abstinent and a lower proportion of heavy drinking days compared in response to NTX versus placebo, whereas participants homozygous for the A allele did not show a significant medication response [93]. Moreover, 87.1% of G allele carriers who received NTX were classified as having a good clinical outcome at study endpoint, versus 54.5% of Asn40 homozygotes who received NTX. (Moderating effects of *OPRM1 *were specific to participants receiving medication management without the cognitive-behavioral intervention [CBI] and were not evident in participants receiving NTX and CBI). A smaller placebo controlled study has also found evidence for better responses to NTX among Asp40 carriers [94]. The Asp40 variant has further been linked to intermediate phenotypes that could influence relapse proneness, including hedonic responses to alcohol [95], increased neural responses to alcohol primes [96], greater craving in response to alcohol use [97] and increased dopamine release in the ventral striatum during alcohol challenge [98]. One study found that the Asp40 allele predicted cue-elicited craving among individuals low in baseline craving but not those high in initial craving, suggesting that tonic craving could interact with genotype to predict phasic responses to drug cues [97].

Findings concerning possible genetic moderators of response to acamprosate have been reported [99], but are preliminary. Additionally, other findings suggest the influence of a *DRD4 *variable number of tandem repeats (VNTR) polymorphism on response to olanzapine, a dopamine antagonist that has been studied as an experimental treatment for alcohol problems. Olanzapine was found to reduce alcohol-related craving those with the long-repeat VNTR (*DRD4 L)*, but not individuals with the short-repeat version (*DRD4 S*; [100,101]). Further, a randomized trial of olanzapine led to significantly improved drinking outcomes in *DRD4 L *but not *DRD4 S *individuals [100].

There is also preliminary evidence for the possibility of genetic influences on response to psychosocial interventions, including those incorporating RP strategies. In a secondary analysis of the Project MATCH data, researchers evaluated posttreatment drinking outcomes in relation to a *GABRA2 *variant previously implicated in the risk for alcohol dependence [102]. Analyses included MATCH participants of European descent who provided a genetic sample (n = 812). Those carrying the high-risk *GABRA2 *allele showed a significantly increased likelihood of relapse following treatment, including a twofold increase in the likelihood of heavy drinking. Furthermore, *GABRA2 *interacted with treatment condition to influence drinking outcomes. Among those with the high-risk genotype, drinking behavior did not appear to be modified by treatment, with outcomes being similar regardless of treatment condition. However, treatment differences emerged in the low-risk genotype group, such that TSF produced the best outcomes, followed by MET [102]. In another psychosocial treatment study, researchers in Poland examined genetic moderators of relapse following inpatient alcohol treatment [103]. Results showed that polymorphisms in *BDNF *(Val66Met) and *COMT *(Val158Met) significantly predicted relapse probability. Overall, evidence for genetic moderation effects in psychosocial trials are consistent with the notion that variants with broad implications for neurotransmitter function, cognitive function, and/or externalizing traits can potentially influence relapse proneness. In the absence of a plausible biological mechanism for differential response to specific psychosocial treatments (e.g., MET vs. CBT) as a function of genotype, the most parsimonious interpretation of these findings is that some variants will impose greater risk for relapse following any quit attempt, regardless of treatment availability or modality.

Findings from numerous non-treatment studies are also relevant to the possibility of genetic influences on relapse processes. For instance, genetic factors could influence relapse in part via drug-specific cognitive processes. Recent studies have reported genetic associations with alcohol-related cognitions, including alcohol expectancies, drinking refusal self-efficacy, drinking motives, and implicit measures of alcohol-related motivation [51,52,104-108]. Overall, the body of research on genetic influences on relapse and related processes is nascent and virtually all findings require replication. Consistent with the broader literature, it can be anticipated that most genetic associations with relapse outcomes will be small in magnitude and potentially difficult to replicate. Nonetheless, initial studies have yielded intriguing results. It is inevitable that the next decade will see exponential growth in this area, including greater use of genome-wide analyses of treatment response [109] and efforts to evaluate the clinical utility and cost effectiveness of tailoring treatments based on pharmacogenetics. Finally, an intriguing direction is to evaluate whether providing clients with personalized genetic information can facilitate reductions in substance use or improve treatment adherence [110,111].

### Mindfulness-based relapse prevention

In terms of clinical applications of RP, the most notable development in the last decade has been the emergence and increasing application of Mindfulness-Based Relapse Prevention (MBRP) for addictive behaviors [112,113]. Given supportive data for the efficacy of mindfulness-based interventions in other behavioral domains, especially in prevention of relapse of major depression [114], there is increasing interest in MBRP for addictive behaviors. The merger of mindfulness and cognitive-behavioral approaches is appealing from both theoretical and practical standpoints [115] and MBRP is a potentially effective and cost-efficient adjunct to CBT-based treatments. In contrast to the cognitive restructuring strategies typical of traditional CBT, MBRP stresses nonjudgmental attention to thoughts or urges. From this standpoint, urges/cravings are labeled as transient events that need not be acted upon reflexively. This approach is exemplified by the "urge surfing" technique [115], whereby clients are taught to view urges as analogous to an ocean wave that rises, crests, and diminishes. Rather than being overwhelmed by the wave, the goal is to "surf" its crest, attending to thoughts and sensations as the urge peaks and subsides.

Results of a preliminary nonrandomized trial supported the potential utility of MBRP for reducing substance use. In this study incarcerated individuals were offered the chance to participate in an intensive 10-day course in Vipassana meditation (VM). Those participating in VM were compared to a treatment as usual (TAU) group on measures of post-incarceration substance use and psychosocial functioning. Relative to the TAU group, the VM group reported significantly lower levels of substance use and alcohol-related consequences and improved psychosocial functioning at follow-up [116].

More recently, a randomized controlled trial compared an eight-week MBRP course to treatment as usual (TAU), which consisted of 12-step-based process-oriented discussion and psychoeducation groups [117]. The majority of MBRP participants (86%) engaged in meditation practices immediately posttreatment and 54% continued practice for at least 4 months posttreatment (M = 4.74 days/week, up to 30 min/day). Compared to TAU, MBRP participants reported significantly reduced craving, and increased acceptance and mindful awareness over the 4-month follow-up period, consistent with the core goals of MBRP. Over the course of treatment, MBRP evinced fewer days of use compared to TAU (MBRP: M = .06 days, TAU: M = 2.57 days). These differences persisted at 2-month follow-up (2.08 days for MBRP vs. 5.43 days for TAU). Secondary analyses [118] showed that compared to TAU, MBRP participants evinced a decreased relation between depressive symptoms and craving following treatment. This attenuation was related to subsequent decreases in alcohol and other drug use, suggesting MBRP led to decreased craving in response to negative affect, thereby lessening the need to alleviate affective discomfort with alcohol and other drug use. Furthermore, individuals with moderate depression in the MBRP group had a significantly lower probability of substance use, fewer drinks per drinking day, and fewer drinks per day than individuals with moderate depression in TAU. A larger, randomized trial comparing MBRP to TAU and RP is currently underway at the University of Washington to evaluate whether the addition of mindfulness to the standard RP treatment leads to better substance use outcomes following treatment. As is the case in other clinical domains [114], interest in MBRP for substance use disorders is increasing rapidly. Results of additional randomized controlled trials will be important for informing its broader application for various addictive behaviors.

## Critiques of the RP Model

Following the initial introduction of the RP model in the 1980s, its widespread application largely outpaced efforts to systematically validate the model and test its underlying assumptions. Given this limitation, the National Institutes on Alcohol Abuse and Alcoholism (NIAAA) sponsored the Relapse Replication and Extension Project (RREP), a multi-site study aiming to test the reliability and validity of Marlatt's original relapse taxonomy. Efforts to evaluate the validity [119] and predictive validity [120] of the taxonomy failed to generate supportive data. It was noted that in focusing on Marlatt's relapse taxonomy the RREP did not comprehensive evaluation of the full RP model [121]. Nevertheless, these studies were useful in identifying limitations and qualifications of the RP taxonomy and generated valuable suggestions [121].

The recently introduced dynamic model of relapse [8] takes many of the RREP criticisms into account. Additionally, the revised model has generated enthusiasm among researchers and clinicians who have observed these processes in their data and their clients [122,123]. Still, some have criticized the model for not emphasizing interpersonal factors as proximal or phasic influences [122,123]. Other critiques include that nonlinear dynamic systems approaches are not readily applicable to clinical interventions [124], and that the theory and statistical methods underlying these approaches are esoteric for many researchers and clinicians [14]. Rather than signaling weaknesses of the model, these issues could simply reflect methodological challenges that researchers must overcome in order to better understand dynamic aspects of behavior [45]. Ecological momentary assessment [44], either via electronic device or interactive voice response methodology, could provide the data necessary to fully test the dynamic model of relapse. Ideally, assessments of coping, interpersonal stress, self-efficacy, craving, mood, and other proximal factors could be collected multiple times per day over the course of several months, and combined with a thorough pre-treatment assessment battery of distal risk factors. Future research with a data set that includes multiple measures of risk factors over multiple days could also take advantage of innovative modeling tools that were designed for estimating nonlinear time-varying dynamics [125].

## Directions for Future Research

Considering the numerous developments related to RP over the last decade, empirical and clinical extensions of the RP model will undoubtedly continue to evolve. In addition to the recent advances outlined above, we highlight selected areas that are especially likely to see growth over the next several years.

### Mechanisms of treatment effects

Elucidating the "active ingredients" of CBT treatments remains an important and challenging goal. Consistent with the RP model, changes in coping skills, self-efficacy and/or outcome expectancies are the primary putative mechanisms by which CBT-based interventions work [126]. However, few studies support these presumptions. One study, in which substance-abusing individuals were randomly assigned to RP or twelve-step (TS) treatments, found that RP participants showed increased self-efficacy, which accounted for unique variance in outcomes [69]. In a recent study, Witkiewitz and colleagues (under review) found that individuals in the combined behavioral intervention of the COMBINE study who received drink-refusal skills training as part of the behavioral intervention had significantly better outcomes than those who did not receive the drink-refusal skills training, particularly African American clients [127]. Further, there was strong support that increases in self-efficacy following drink-refusal skills training was the primary mechanism of change. In another study examining the behavioral intervention arm of the COMBINE study [128], individuals who received a skills training module focused on coping with craving and urges had significantly better drinking outcomes via decreases in negative mood and craving that occurred after receiving the module.

Despite findings like these, many studies of treatment mechanisms have failed to show that theoretical mediators account for salutary effects of CBT-based interventions. Also, many studies that have examined potential mediators of outcomes have not provided a rigorous test [129] of mechanisms of change. These results suggest that researchers should strive to consider alternative mechanisms, improve assessment methods and/or revise theories about how CBT-based interventions work [77,130].

### Continued empirical evaluation of the RP model

As the foregoing review suggests, validation of the reformulated RP model will likely progress slowly at first because researchers are only beginning to evaluate dynamic relapse processes. Currently, the dynamic model can be viewed as a hypothetical, theory-driven framework that awaits empirical evaluation. Testing the model's components will require that researchers avail themselves of innovative assessment techniques (such as EMA) and pursue cross-disciplinary collaboration in order to integrate appropriate statistical methods. Irrespective of study design, greater integration of distal and proximal variables will aid in modeling the interplay of tonic and phasic influences on relapse outcomes. As was the case for Marlatt's original RP model, efforts are needed to systematically evaluate specific theoretical components of the reformulated model [1].

### Integrating implicit cognition and neurocognition in relapse models

Historically, cognitive processes have been central to the RP model [8]. In the last several years increasing emphasis has been placed on "dual process" models of addiction, which hypothesize that distinct (but related) cognitive networks, each reflective of specific neural pathways, act to influence substance use behavior. According to these models, the relative balance between *controlled *(explicit) and *automatic *(implicit) cognitive networks is influential in guiding drug-related decision making [54,55]. Dual process accounts of addictive behaviors [56,57] are likely to be useful for generating hypotheses about dynamic relapse processes and explaining variance in relapse, including episodes of sudden divergence from abstinence to relapse. Implicit cognitive processes are also being examined as an intervention target, with some potentially promising results [62].

Related work has also stressed the importance of baseline levels of neurocognitive functioning (for example as measured by tasks assessing response inhibition and working memory; [56]) as predicting the likelihood of drug use in response to environmental cues. The study of implicit cognition and neurocognition in models of relapse would likely require integration of distal neurocognitive factors (e.g., baseline performance in cognitive tasks) in the context of treatment outcomes studies or EMA paradigms. Additionally, lab-based studies will be needed to capture dynamic processes involving cognitive/neurocognitive influences on lapse-related phenomena.

### Evaluating neural markers of relapse liability

The use of functional magnetic resonance imaging (fMRI) techniques in addictions research has increased dramatically in the last decade [131] and many of these studies have been instrumental in providing initial evidence on neural correlates of substance use and relapse. In one study of treatment-seeking methamphetamine users [132], researchers examined fMRI activation during a decision-making task and obtained information on relapse over one year later. Based on activation patterns in several cortical regions they were able to correctly identify 17 of 18 participants who relapsed and 20 of 22 who did not. Functional imaging is increasingly being incorporated in treatment outcome studies (e.g., [133]) and there are increasing efforts to use imaging approaches to predict relapse [134]. While the overall number of studies examining neural correlates of relapse remains small at present, the coming years will undoubtedly see a significant escalation in the number of studies using fMRI to predict response to psychosocial and pharmacological treatments. In this context, a critical question will concern the predictive and clinical utility of brain-based measures with respect to predicting treatment outcome.

## Conclusions and Policy Implications

Relapse prevention is a cognitive-behavioral approach designed to help individuals anticipate and cope with setbacks during the behavior change process. The broad aim of RP, to reduce the incidence and severity of relapse, subsumes two basic goals: to minimize the impact of high-risk situations by increasing awareness and building coping skills, and to limit relapse proneness by promoting a healthy and balanced lifestyle. Over the past decade RP principles have been incorporated across an increasing array of behavior domains, with addictive behaviors continuing to represent the primary application.

As outlined in this review, the last decade has seen notable developments in the RP literature, including significant expansion of empirical work with relevance to the RP model. Overall, many basic tenets of the RP model have received support and findings regarding its clinical effectiveness have generally been supportive. RP modules are standard to virtually all psychosocial interventions for substance use [17] and an increasing number of self-help manuals are available to assist both therapists and clients. RP strategies can now be disseminated using simple but effective methods; for instance, mail-delivered RP booklets are shown to reduce smoking relapse [135,136]. As noted earlier, the broad influence of RP is also evidenced by the current clinical vernacular, as "relapse prevention" has evolved into an umbrella term synonymous with most cognitive-behavioral skills-based interventions addressing high-risk situations and coping responses. While attesting to the influence and durability of the RP model, the tendency to subsume RP within various treatment modalities can also complicate efforts to systematically evaluate intervention effects across studies (e.g., [21]).

Although many developments over the last decade encourage confidence in the RP model, additional research is needed to test its predictions, limitations and applicability. In particular, given recent theoretical revisions to the RP model, as well as the tendency for diffuse application of RP principles across different treatment modalities, there is an ongoing need to evaluate and characterize specific theoretical mechanisms of treatment effects.

In the current review we have noted several areas for future research, including examining dynamic models of treatment outcomes, extensions of RP to include mindfulness and/or self-control training, research on the mechanisms of change following successful treatment outcomes, the role of genetic influences as potential moderators of treatment outcomes, and neurocognitive and neurobiological examinations of the relapse process using tests of implicit cognition and advanced neuroimaging techniques. In addition to these areas, which already have initial empirical data, we predict that we could learn significantly more about the relapse process using experimental manipulation to test specific aspects of the cognitive-behavioral model of relapse. For example, it has been shown that self-efficacy for abstinence can be manipulated [137]. Thus, one could test whether increasing self-efficacy in an experimental design is related to better treatment outcomes. Similarly, self-regulation ability, outcome expectancies, and the abstinence violation effect could all be experimentally manipulated, which could eventually lead to further refinements of RP strategies.

Ultimately, individuals who are struggling with behavior change often find that making the initial change is not as difficult as maintaining behavior changes over time. Many therapies (both behavioral and pharmacological) have been developed to help individuals cease or reduce addictive behaviors and it is critical to refine strategies for helping individuals maintain treatment goals. As noted by McLellan [138] and others [124], it is imperative that policy makers support adoption of treatments that incorporate a continuing care approach, such that addictions treatment is considered from a chronic (rather than acute) care perspective. Broad implementation of a continuing care approach will require policy change at numerous levels, including the adoption of long-term patient-based and provider-based strategies and contingencies to optimize and sustain treatment outcomes [139,140].

In support of continuing care approaches the United States Office of National Drug Control Policy recently published the 2010 National Drug Control Strategy in the United States [141], which includes strategies to integrate treatment for substance use disorders into the mainstream health care system and to expand support for continuing care efforts. One critical goal will be to integrate empirically supported substance use interventions in the context of continuing care models of treatment delivery, which in many cases requires adapting existing treatments to facilitate sustained delivery [140]. Given its focus on long-term maintenance of treatment gains, RP is a behavioral intervention that is particularly well suited for implementation in continuing care contexts. Many treatment centers already provide RP as a routine component of aftercare programs. However, it is imperative that insurance providers and funding entities support these efforts by providing financial support for aftercare services. It is also important that policy makers and funding entities support initiatives to evaluate RP and other established interventions in the context of continuing care models. In general, more research on the acquisition and long-term retention of specific RP skills is necessary to better understand which RP skills will be most useful in long-term and aftercare treatments for addictions.

## Competing interests

The authors declare that they have no competing interests.

## Authors' contributions

CH wrote the manuscript with contributions from KW. BG and GAM assisted in conceptualizing the paper and provided critical review. All authors read and approved the manuscript.
